# Cardiovascular Health in India – a Report Card from Three Urban and Rural Surveys of 22,144 Adults

**DOI:** 10.5334/gh.1137

**Published:** 2022-08-02

**Authors:** Roopa Shivashankar, Kalpana Singh, Dimple Kondal, Ruby Gupta, Pablo Perel, Deksha Kapoor, Devraj Jindal, Sailesh Mohan, Rajendra Pradeepa, Prashant Jarhyan, Nikhil Srinivasapura Venkateshmurthy, Nikhil Tandon, Viswanathan Mohan, K. M. Venkat Narayan, Dorairaj Prabhakaran, Mohammed K. Ali

**Affiliations:** 1 Indian Council of Medical Research (ICMR), New Delhi, IN; 2 Centre for Chronic Disease Control (CCDC), New Delhi, IN; 3 Hamad Medical Corporation, Doha, QA; 4 Public Health Foundation of India (PHFI), New Delhi, IN; 5 London School of Hygiene and Tropical Medicine (LSHTM), London, UK; 6 All India Institute of Medical Sciences (AIIMS), New Delhi, IN; 7 Global Academy of Agriculture and Food Systems, University of Edinburgh, Edinburgh, UK; 8 Deakin University, Melbourne, AUS; 9 Madras Diabetes Research Foundation (MDRF), Chennai, IN; 10 Rollins School of Public Health & Emory Global Diabetes Research Center, Emory University, Atlanta, US; 11 Rollins School of Public Health, Emory University, Atlanta, US; 12 Rollins School of Public Health & Department of Family and Preventive Medicine, School of Medicine, Emory University, Atlanta, US

**Keywords:** Cardiovascular health, urban living, socioeconomic status, India, Population Survey

## Abstract

**Background:**

Markers of ideal cardiovascular health (CVH) predict cardiovascular events. We estimated the prevalence of ideal CVH markers in two levels of cities and villages in India.

**Methods:**

We did pooled analysis of individual-level data from three cross sectional surveys of adults ≥ 30 years over 2010–14 (CARRS: Centre for cArdiometabolic Risk Reduction in South Asia; UDAY and Solan Surveillance Study) representing metropolitan cities; smaller cities and rural areas in diverse locations of India. We defined ideal CVH using modified American Heart Association recommendations: not smoking, ≥ 5 servings of fruits and vegetables (F&V), high physical activity (PA), body mass index (BMI) <25 Kg/m^2^, blood pressure (BP) <120/80 mm Hg, fasting plasma glucose (FPG) <100 mg/dl, and total cholesterol (TC) <200 mg/dL. We estimated (1) age-and sex-standardized prevalence of ideal CVH and (2) prevalence of good (≥6 markers), moderate (4–5), and poor CVH (≤3) adjusted for age, sex, education, and stratified by setting and asset tertiles.

**Results:**

Of the total 22,144 participants, the prevalence of ideal CVH markers were: not smoking (76.7% [95% CI 76.1, 77.2]), consumed ≥5 F&V (4.2% [3.9, 4.5]), high PA (67.5% [66.8, 68.2]), optimum BMI (59.6% [58.9, 60.3]), ideal BP (34.5% [33.9, 35.2]), FPG (65.8% [65.1, 66.5]) and TC (65.4% [64.7, 66.1]). The mean number of ideal CVH metrics was 3.7(95% CI: 3.7, 3.8). Adjusted prevalence of good, moderate, and poor CVH, varied across settings: metropolitan (3.9%, 41.0%, and 55.1%), smaller cities (7.8%, 49.2%, and 43%), and rural (10.4%, 60.9%, and 28.7%) and across asset tertiles: Low (11.0%, 55.9%, 33.1%), Middle (6.3%, 52.2%, 41.5%), and High (5.0%, 46.4%, 48.7%), respectively.

**Conclusion:**

Achievement of ideal CVH varied, with higher prevalence in rural and lower asset tertiles. Multi-sectoral and targeted policy and program actions are needed to improve CVH in diverse contexts in India.

## Introduction

Cardiovascular diseases (CVD) are the most common causes of mortality and morbidity globally, and disproportionate part of the CVD burden are experienced by low and middle-income countries (LMICs) like India [1,2]. The Interheart study (2004), a case-control study of first-onset acute myocardial infarctions in 52 countries, attributed 90% of first myocardial infarctions to nine risk factors, most of which were modifiable [3]. In 2010, the American Heart Association (AHA) proposed that seven ‘ideal cardiovascular health (CVH)’ markers –which include: not smoking; high physical activity; healthful diets; body mass index (BMI) <25 Kg/m^2^; blood pressure (BP) <120/80 mm Hg; fasting plasma glucose (FPG) <100; and total cholesterol (TC) <200 mg/dl– would offer a focused set of indicators to assess cardiovascular health in populations [4].

India is at the epicentre of CVD, but little is known about ideal CVH [5,6]. Large numbers in the population are moving from rural areas to cities and towns due to economic transition [7]. CVH metrics will likely differ across and within urban and rural settings in India, but no previous data has explored this. In a recent global systematic review on the prevalence of ideal CVH, only one out of 88 studies included was from India [8]. The study in question [9] included data from 11 small or medium-sized cities and did not capture the variation in urban and rural settings. To address these gaps, we compiled data from three large household surveys from different cities and villages. All three studies used similar data collection methods and occurred during a comparable timeframe. We examined the variation in CVH markers by age, sex, and socioeconomic status in metropolitan and small cities and rural parts of North and South India. These data on metrics for ideal CVH can stimulate policy action, and serve as a benchmark to measure progress.

## Methods

### Data

We pooled individual-level cross-sectional data from three surveys in India that collected data on CVD risk factors between 2010 and 2014. The studies included were the Centre for Cardiometabolic Risk Reduction in South Asia study (CARRS) [10], Comprehensive Diabetes Prevention and Management Program study (UDAY) [11], and the Solan Surveillance Study (SSS) [12]. In the CARRS, adults ≥20 years in two metropolitan cities (Chennai in South India and Delhi in North India) were recruited through sex stratified multistage cluster sampling to ensure representativeness during 2010–11. In the UDAY, adults ≥30 years were recruited by a sex-stratified multistage cluster sampling method from urban and rural areas of the district of Sonipat (Haryana, North India) and Visakhapatnam (Andhra Pradesh, South India) during 2014–15. In SSS, all consenting adults ≥20 years in 38 health sub-centre population of Solan district in Himachal Pradesh, North India, were assessed for cardiometabolic diseases and risk factors using questionnaires over 2012–14. Data collection was similar for all three studies. However, venous fasting blood samples were taken only in a sub-sample in SSS. In this analysis, we included subjects with age ≥30 years who had fasting blood samples.

### Data collection

Details and comparisons of settings, study designs, participant recruitment approaches, and data collection tools are provided in Table 1. In all three studies, information on sociodemographic details, cardiac risk behaviours and factors, and medical history were obtained through interviewer-administered questionnaires, measurements, and fasting blood samples. The UDAY and SSS adopted the same questions used in the CARRS study, and therefore the data were comparable. The data collectors of all three studies had been trained by the central teams at the Centre for Chronic Disease Control (CCDC) and Public Health Foundation of India (PHFI). The blood samples were analyzed at the PHFI laboratory for glucose and cholesterol for all sites except for Chennai. The Chennai samples were analyzed at the Madras Diabetes Research Foundation (MDRF) laboratory. Both PHFI and MDRF laboratories analysed the samples using similar methods for plasma glucose and total cholesterol. They also participated and passed the external quality assurance program through Randox international quality assessment scheme (RIQAS).

**Table 1:** Comparison of setting, designs, participant recruitment and data collection of CARRS, UDAY and SSS.


	CARRS		UDAY				SSS
		
**Setting**	North	South	North		South		North

**Residence**	Metropolitan cities		Smaller city	Rural	Smaller city	Rural	Rural

**District**	Delhi	Chennai	Sonipat		Vishakhapatnam		Solan

**Household sampling**	Multistage cluster random						Volunteer sampling

**Individual sampling**	1 man and 1 woman from each household (selected by Kish method)						

**Age groups**	≥20 years		≥30 years				≥20 years

**Exclusion criteria**	Pregnant, bedridden and participants who were unable to comprehend the questionnaires due cognitive deficiencies were excluded						

**Study period**	October 2010 – November 2011		July 2014 – February 2015				2013–14

**Laboratory**	PHFI	MDRF	PHFI				PHFI

**Data collection tools**							

**Age, sex, education, household assets**	Questionnaire						

**Current Smoking**	Questionnaire- Self-reported use of smoked tobacco- ever and current						

**Physical activity**	International Physical Activity Questionnaire – Short Form (IPAQ-SF)		Global Physical Activity Questionnaire (GPAQ)				IPAQ-SF

**Fruits and vegetables servings**	Food frequency questionnaire (FFQ)		WHO STEPS questions on fruit and vegetable intake				FFQ

**Blood pressure**	Omron HEM – 7080;		Omron HEM – 7200				Omron T9P

**BMI**	Weight – Tanita – BC 601; Height – Seca 214 stadiometer						Weight – Omron 286; Height – Seca 214 stadiometer

**Fasting plasma glucose**	Hexokinase/Kinetic						

**Total Cholesterol**	Cholesterol Oxidase Peroxidase (CHOD-POD) end-point						


*Notes*: Abbreviations CARRS- Centre for Cardiometabolic Risk Reduction in South Asia; UDAY- Comprehensive Diabetes Prevention and Management Program study; SSS- Solan Surveillance study; PHFI- Public Health Foundation of India; MDRF- Madras Diabetes Research Foundation; WHO-STEPS- World Health Organization- STEPwise approach to surveillance.

### Variables

We harmonized the variables from the three surveys. We categorized age into three age groups: 30–44, 45–59, and 60+ years. We categorized self-reported education levels into three categories – primary schooling; high or secondary schooling; and university or higher. We used the information on the presence of various household amenities (separate cooking room and toilet facilities) and assets (television, refrigerator, mobile phone, computer, car, motorcycle, and bicycle) to derive an asset index using principal components analysis [13]. Total asset scores were categorized into tertiles (lowest tertile representing poorest and highest tertile representing wealthiest).

For individual CVH markers, we modified the AHA definitions slightly. First, we stratified CVH markers disregarding use of medications to control hypertension, diabetes, and dyslipidemia; this is because the interventions to improve cardiovascular health includes medication for those with diagnosed conditions and assigning all participants on treatment to poor CVH would exclude those lowering their CVD risk with appropriate pharmacotherapies. As a sensitivity analysis, we conducted the analysis using the original AHA definitions wherein all pharmacotherapies were allocated to poor CVH. Second, we used fruits and vegetable consumption as a marker of healthful diet as other dietary variables were not available in all the three surveys included.

Details of the individual ideal CVH markers are provided below.

1. Smoking: Self-reported tobacco smoking was used to code the participant as a non-smoker (ideal), stopped smoking <6 months (intermediate), and current smoker (poor).
2. Diet: average daily servings of fruits and vegetables consumed was classified as ≥5 (ideal), 2–4 (intermediate), and <2 (poor).
3. Physical activity: We converted the information on physical activity from the International Physical Activity Questionnaire-short form (IPAQ-SF) [14] and Global Physical Activity Questionnaire (GPAQ) [15] into metabolic equivalent minutes (Met minutes) using standard guidelines and stratified participants with high, moderate, and low activity as ideal, intermediate, and poor, respectively [15,16].
4. BMI: Measured height in metres and weight in Kilograms were used to calculate BMI. The cut-offs for BMI for ideal, intermediate, and poor categories were <25.0, 25–29.9, and ≥30.0 Kg/m^2^, respectively.
5. Blood Pressure (BP): BP was measured in sitting position after five-minute rest. Up to three readings were taken with a gap of at least 30 seconds. The average of the last two readings was used. The cut-offs for ideal, intermediate, and poor BP were <120/80, 120–139/80–89, and ≥140/90 mm Hg, respectively.
6. Fasting Plasma Glucose (FPG): Measured from fasting blood samples, we categorized levels as <100 mg/dl (ideal), 100–125 mg/dl (intermediate), and ≥126 mg/dl (poor), respectively.
7. Total Cholesterol (TC): Measured from fasting blood samples, we categorized ideal, intermediate, and poor levels as <200, 200–239 and >= 240 mg/dl, respectively.

We further grouped participants with ≥6, 4 to 5, and ≤3 ideal CVH metrics as having good, moderate, and poor CVH, respectively.

### Ethical considerations

The CARRS was approved by the institutional review boards (IRBs) of the PHFI, MDRF and Emory University. The UDAY and SSS were approved by IRB’s of PHFI and CCDC, respectively. Informed consent was obtained by all the participants of the three surveys and deidentified data were analysed.

### Analysis

**Figure 1:**
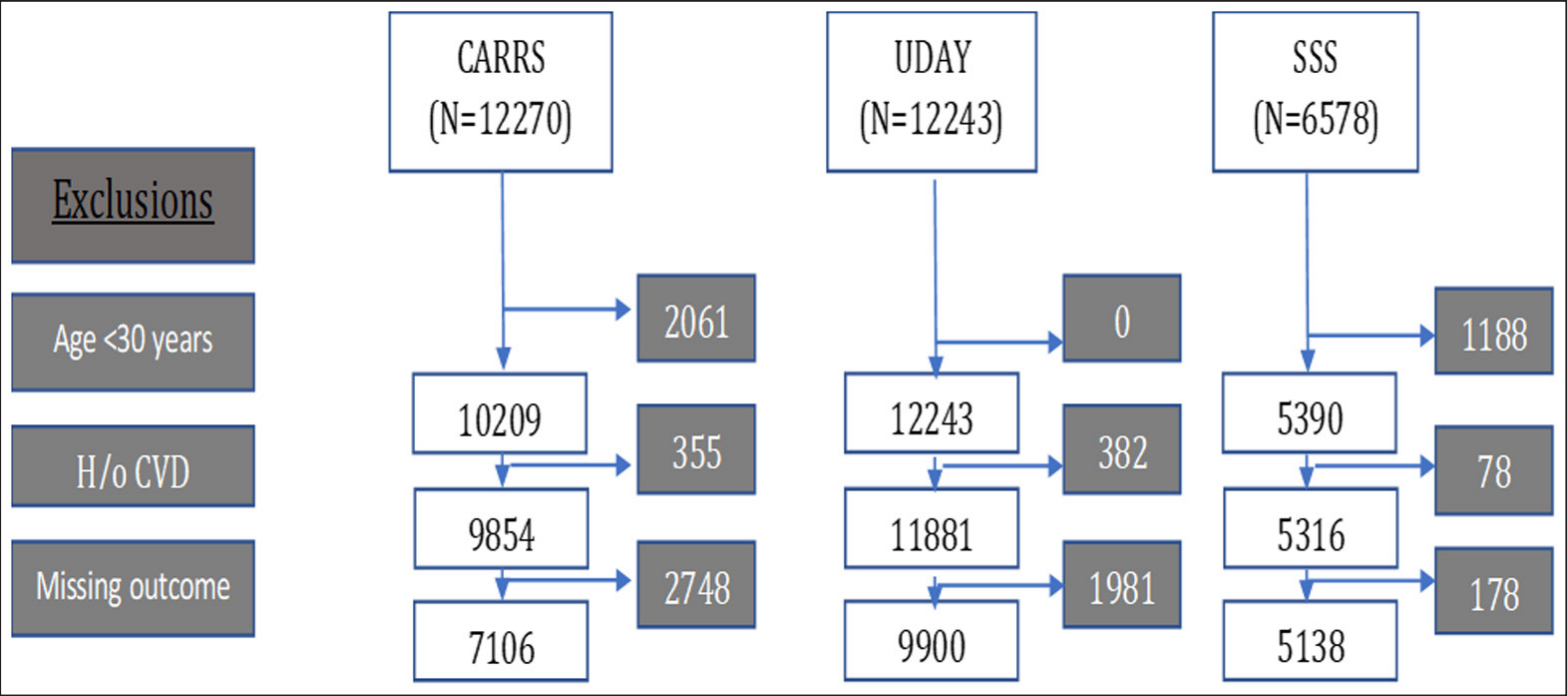
Flow chart of participant selections from CARRS, UDAY, and SSS. *Notes*: Abbreviations CARRS- Centre of Cardiometabolic Risk Reduction in South Asia; SSS- Solan Surveillance Study; h/o CVD- history of CVD. * Missing outcome- If data is missing on one or more of tobacco use, diet, physical activity, blood pressure, body mass index, fasting plasma glucose or total cholesterol.

The total sample size from the three studies were 31,091 (CARRS – 12270, UDAY – 12,243 and SSS – 6,578). We excluded those who were <30 years (excluded 8,967), reported prior cardiac disease or stroke (excluded 791), and those with incomplete data for the seven outcome variables of interest (excluded 4,907) (Figure 1). The final sample size for current analysis was 22,144: metropolitan = 7,106 (Chennai – 3,858, Delhi – 3,248), smaller cites = 4,948 (Sonipat – 2,536, Visakhapatnam – 2412), and rural = 10,090 (Sonipat– 2,299, Visakhapatnam – 2,653, and Solan 5,138).

We used Stata 14.2 for all analyses. We accounted for the complex survey design in two surveys, that is, CARRS and UDAY for analyses by adjusting standard errors for clustering and incorporating sampling weights. We used a standard weight of 1 for the SSS study data. All estimates were age- and sex-standardized to the 2010 South Asia regional population [16].

We described (percentages or means with 95% confidence interval) the sociodemographic characteristics (age, sex, education, and asset index) by metropolitan, smaller city, and rural settings. We estimated both crude and age-sex standardized prevalence of ideal CVH metrics, separately and combined into good, moderate, and poor overall CVH markers.

To explore the heterogeneity across India, we estimated crude and adjusted prevalence (average predicted marginals estimated from the polytomous logistic regression models comparing good and moderate CVH against the poor CVH) of good, moderate, and poor CVH across all settings combined and then by site (metropolitan vs. smaller cities vs. rural), age groups (30–44 vs. 45–59 vs. ≥60 years), sex (men vs. women), education (up to primary vs. secondary school vs. graduates plus) and asset tertiles (low vs. middle and high).

Finally, we performed sensitivity analyses to assess the impact missing data and modified definition of ideal CVH. First, we compared the demographics (age, sex, SES, and education) of the complete case data with data including those had missing variables in cardiac risk factors for total dataset, and separately for CARRS, Uday, and SSS datasets. Second, we estimated the percentages and means of ideal cardiac metrics using inverse probability weighting approach. [17] That is, we weighted analysis by inverse of predictive probability of ideal cardiac health metrics computed based on the sociodemographic variables – age, sex, education and wealth index. Third, we reclassified those who were using medications for diabetes, hypertension, and/or dyslipidemia as ‘poor’ CVH and compared the proportion of ideal CVH with the earlier classification based on measured values that disregarded treatment status.

## Results

Demographic characteristics were different across settings (Table 2). Overall, the mean (SD) age of the participants was 46.8 (11.9) years. Women constituted 56% of the study population. Graduates constituted 14.4% and low, middle, and high asset tertiles accounted for 34.2%, 36.5%, and 29.4% of the population, respectively.

**Table 2:** Sociodemographic characteristics of study population free of cardiovascular diseases.


VARIABLES	METROPOLITAN CITIES	SMALLER CITIES	RURAL	TOTAL	P-VALUE*

	n = 7106	n = 4948	n = 10,090	n = 22,144	

	COL % (95% CI)	COL % (95% CI)	COL % (95% CI)	COL % (95% CI)	

Age Mean (SD)	45.7 (11.1)	46.9 (12.1)	47.5 (12.3)	46.8 (11.9)	<0.001

Age group					

**30–44**	51.3 (50.1, 52.5)	47.5 (46.1, 48.9)	45.7 (44.7, 46.6)	47.9 (47.2, 48.5)	**<0.001**

**45–60**	34.8 (33.7, 35.9)	34.7 (33.4, 36.1)	33.9 (33.0, 34.8)	34.4 (33.7, 35.0)	

**>60**	13.9 (13.1, 14.8)	17.8 (16.7, 18.9)	20.5 (19.7, 21.3)	17.8 (17.3, 18.3)	

Sex					

**Men**	45.2 (44.1, 46.4)	46.3 (44.9, 47.7)	42.1 (41.2, 43.1)	44.0 (43.4, 44.7)	**<0.001**

**Women**	54.8 (53.6, 55.9)	53.7 (52.3, 55.1)	57.9 (56.9, 58.8)	56.0 (55.3, 56.6)	

Education					

**Up to primary schooling**	20.3 (19.4, 21.3)	24.8 (23.6, 26.1)	38.9 (37.9, 39.8)	29.8 (29.2, 30.4)	**<0.001**

**Secondary school**	63.2 (62.1, 64.4)	47.6 (46.2, 49.0)	54.6 (53.6, 55.6)	55.8 (55.2, 56.5)	

**Graduation and above**	16.4 (15.6, 17.3)	27.5 (26.3, 28.8)	6.5 (6.1, 7.0)	14.4 (13.9, 14.9)	

Asset tertile					

**Low**	38.2 (37.0, 39.3)	20.0 (18.9, 21.1)	38.8 (37.7, 39.8)	34.2 (33.5, 34.8)	**<0.001**

**Middle**	37.0 (35.9, 38.1)	36.9 (35.6, 38.3)	35.8 (34.8, 36.8)	36.5 (35.8, 37.1)	

**High**	24.8 (23.8, 25.8)	43.1 (41.7, 44.5)	25.5 (24.6, 26.4)	29.4 (28.8, 30.0)	


*Notes*: Metropolitan cities- Delhi and Chennai; Smaller cities- urban Sonipat and Visakhapatnam; Rural- rural areas of Sonipat, Visakhapatnam and Solan; Abbreviations: SD- standard deviation. * Using chi-square (for percentages) or one-way ANOVA (for means) tests.

The crude and age-sex standardized prevalence of ideal, intermediate, and poor levels of individual cardiac health metrics varied by setting (Table 3). Age and sex standardized prevalence of non-smokers was 76.7% overall, with higher levels in metropolitan cities (84.2%) and smaller cities (80.3%) compared to rural areas (70.5%). Only 4.2% reported consuming ≥5 servings of fruits and vegetables a day which varied from 0.4% in metropolitan to 11.4% in smaller cities. Ideal physical activity levels were reported by 67.5 overall, which varied from 53.9% in smaller cities to 78.7% in rural areas. Ideal BP was observed in 34.5%, which was lowest in metropolitan cities (28.5%) and highest in rural villages (38.5%). The prevalence of ideal BMI and FPG were 59.6% and 65.8%, respectively, which was lowest in metropolitan cities (47.0%) and (48.1%) and highest in rural areas (73.3%) and (77.9%), respectively. Ideal cholesterol levels were found in 65.4%, which did not vary by setting.

**Table 3:** Distribution of individual ideal cardiac health metrics in the study population free from cardiovascular diseases.


VARIABLES	DEFINITION	METROPOLITAN CITIES		SMALLER CITIES		RURAL		TOTAL		
			
		n = 7,106		n = 4,948		n = 10,090		n = 22,144		
			
		CRUDE	STANDARDIZED*	CRUDE	STANDARDIZED*	CRUDE	STANDARDIZED*	CRUDE	STANDARDIZED*	P-VALUE *

Smoking										

**Ideal**	Non-smoker	85.4 (84.5, 86.2)	84.2 (83.3, 85.1)	81.9 (80.8, 82.9)	80.3 (79.2, 81.4)	73.8 (72.9, 74.6)	70.5 (69.6, 71.4)	79.3 (78.8, 79.8)	76.7 (76.1, 77.2)	**<0.001**

**Intermediate**	Past smoker	0	0	0	0	0	0	0	0	

**Poor**	Current smoker	14.6 (13.8, 15.4)	15.8 (14.9, 16.7)	18.1 (17.1, 19.2)	19.7 (18.6, 20.8)	26.2 (25.4, 27.1)	29.5 (28.6, 30.4)	20.7 (20.1, 21.2)	23.3 (22.7, 23.9)	

Diet (fruits and vegetable consumption)										

**Ideal**	≥ 5 servings	0.5 (0.3, 0.7)	0.4 (0.3, 0.6)	11.7 (10.9, 12.7)	11.4 (10.4, 12.3)	3.2 (2.9, 3.6)	3.3 (2.9, 3.7)	4.2 (4.0, 4.5)	4.2 (3.9, 4.5)	**<0.001**

**Intermediate**	2–4 servings	45.6 (44.4, 46.7)	45.7 (44.4, 47.1)	52.0 (50.6, 53.4)	52.0 (50.4, 53.5)	41.6 (40.7, 42.6)	41.4 (40.4, 42.5)	45.2 (44.6, 45.9)	45.1 (44.4, 45.8)	

**Poor**	<2 servings	54.0 (52.8, 55.1)	53.8 (52.5, 55.2)	36.2 (34.9, 37.6)	36.7 (35.2, 38.1)	55.1 (54.2, 56.1)	55.2 (54.2, 56.3)	50.5 (49.9, 51.2)	50.7 (50.0, 51.4)	

**Mean servings**		1.5(1.5, 1.5)	1.5(1.5, 1.5)	2.9 (2.8, 2.9)	2.8 (2.8, 2.9)	2.1 (2.1, 2.1)	2.1 (2.1, 2.1)	2.1 (2.1, 2.1)	2.1 (2.1, 2.1)	<0.001

Physical activity										

**Ideal**	High activity	66.4 (65.3, 67.5)	60.8 (59.5, 62.1)	57.8 (56.5, 59.2)	53.9 (52.4, 55.3)	81.4 (80.6, 82.1)	78.7 (77.8, 79.5)	71.3 (70.7, 71.9)	67.5 (66.8, 68.2)	**<0.001**

**Intermediate**	Moderate activity	25.9 (24.9, 26.9)	29.0 (27.7, 30.2)	26.5 (25.3, 27.8)	27.9 (26.5, 29.3)	10.9 (10.3, 11.6)	12.0 (11.3, 12.7)	19.2 (18.7, 19.7)	20.9 (20.3, 21.5)	

**Poor**	Low activity	7.7 (7.1, 8.4)	10.2 (9.3, 11.1)	15.6 (14.6, 16.7)	18.2 (17.0, 19.4)	7.7 (7.2, 8.2)	9.3 (8.7, 10.0)	9.5 (9.1, 9.9)	11.6 (11.1, 12.1)	

**Mean MET minutes**		4478.0 (4410.7, 4545.2)	4159.1 (4084.0, 4234.2)	5636.0 (5464.6, 5807.4)	5254.9 (5084.0, 5425.9)	9700.3 (9568.1, 9832.6)	9244.7 (9108.5, 9380.9)	7,119.6 (7,038.5, 7,200.7)	6708.3 (6624.2, 6792.4)	<0.001

Blood Pressure (BP in mm/Hg)										

**Ideal**	<120/80	32.2 (31.1, 33.3)	28.5 (27.4, 29.6)	38.2 (36.9, 39.6)	34.5 (33.2, 35.8)	41.4 (40.4, 42.3)	38.5 (37.5, 39.5)	37.7 (37.1, 38.4)	34.5 (33.9, 35.2)	**<0.001**

**Intermediate**	120–139/80–89	38.3 (37.2, 39.5)	37.9 (36.6, 39.3)	36.9 (35.6, 38.3)	37.9 (36.4, 39.4)	38.5 (37.5, 39.4)	39.0 (37.9, 40.0)	38.1 (37.5, 38.7)	38.4 (37.7, 39.1)	

**Poor**	≥140/90	29.4 (28.4, 30.5)	33.6 (32.3, 34.9)	24.9 (23.7, 26.1)	27.6 (26.3, 29.0)	20.1 (19.4, 20.9)	22.5 (21.6, 23.4)	24.2 (23.6, 24.8)	27.1 (26.4, 27.7)	

**Mean Systolic BP**		125.5 (125.0, 125.9)	128.5 (127.9, 129.0)	126.6 (126.0, 127.2)	128.6 (128.0, 129.2)	123.9 (123.5, 124.2)	125.5 (125.1, 125.9)	125.0 (124.7, 125.2)	127 (126.7, 127.3)	

**Mean Diastolic BP**		83.1 (82.9, 83.4)	83.5 (83.1, 83.8)	77.4 (77.1, 77.7)	77.2 (76.8, 77.5)	76.9 (76.7, 77.2)	77.1 (76.8, 77.3)	79.0 (78.9, 79.2)	79.1 (78.9, 79.2)	<0.001

Body mass index (BMI in kg/m2)										

**Ideal**	<25.0	44.4 (43.2, 45.5)	47.0 (45.6, 48.3)	46.7 (45.3, 48.1)	48.2 (46.7, 49.7)	72.1 (71.2, 73.0)	73.3 (72.4, 74.2)	57.5 (56.9, 58.2)	59.6 (58.9, 60.3)	**<0.001**

**Intermediate**	25.0–29.9	36.0 (34.9, 37.1)	34.3 (33.0, 35.6)	36.5 (35.1, 37.8)	35.4 (34.0, 36.9)	21.5 (20.7, 22.3)	20.8 (19.9, 21.6)	29.5 (28.9, 30.1)	28.3 (27.6, 28.9)	

**Poor**	≥ 30	19.6 (18.7, 20.5)	18.7 (17.7, 19.8)	16.8 (15.8, 17.9)	16.4 (15.3, 17.5)	6.4 (5.9, 6.9)	5.9 (5.5, 6.4)	12.9 (12.5, 13.4)	12.1 (11.7, 12.6)	

**Mean BMI**		26.0 (25.9, 26.1)	25.7 (25.6, 25.8)	25.7 (25.6, 25.9)	25.6 (25.4, 25.8)	22.9 (22.8, 23.0)	22.7 (22.7, 22.8)	24.5 (24.4, 24.6)	24.3 (24.2, 24.4)	<0.001

Fasting plasma glucose (FPG in mg/dl)										

**Ideal**	<100	50.0 (48.9, 51.2)	48.1 (46.8, 49.4)	65.6 (64.3, 66.9)	64.4 (63.0, 65.8)	78.4 (77.6, 79.2)	77.9 (77.0, 78.8)	66.5 (65.8,67.1)	65.8 (65.1, 66.5)	**<0.001**

**Intermediate**	100–125	33.0 (31.9, 34.1)	33.1 (31.8, 34.4)	20.7 (19.6, 21.9)	21.0 (19.8, 22.3)	16.3 (15.6, 17.0)	16.5 (15.7, 17.2)	22.7 (22.1,23.2)	22.6 (22.0, 23.3)	

**Poor**	≥126	16.9 (16.1, 17.8)	18.9(17.8, 20.0)	13.7 (12.8, 14.7)	14.6 (13.5, 15.6)	5.3 (4.9, 5.7)	5.7 (5.2, 6.1)	10.9 (10.5, 11.3)	11.6 (11.1, 12.0)	

**Mean FPG**		113.8 (112.7, 114.8)	115.1 (113.8, 116.4)	103.9 (102.7, 105.1)	104.6 (103.3, 106.0)	94.4 (93.9, 95.0)	94.9 (94.2, 95.5)	102.8 (102.2, 103.3)	103.2 (102.6, 103.8)	<0.001

Total Cholesterol (TC in mg/dl)										

**Ideal**	<200	66.1 (65.0, 67.2)	64.1 (62.8, 65.4)	66.2 (64.9, 67.5)	65.8 (64.4, 67.2)	66.5 (65.6, 67.4)	65.9 (65.0, 66.9)	66.3 (65.7, 66.9)	65.4 (64.7, 66.1)	0.07

**Intermediate**	200–239	25.0 (24.0, 26.1)	26.2 (25.0, 27.4)	24.5 (23.3, 25.7)	24.7 (23.4, 26.0)	23.6 (22.8, 24.4)	23.7 (22.8, 24.6)	24.3 (23.7,24.8)	24.7 (24.0, 25.3)	

**Poor**	≥240	8.8 (8.2, 9.5)	9.7 (8.9, 10.6)	9.3 (8.5, 10.2)	9.5 (8.6, 10.4)	9.9 (9.3, 10.5)	10.3 (9.7, 11.0)	9.4 (9.1, 9.8)	10.0 (9.5, 10.4)	

**Mean TC**		186.5 (185.6, 187.4)	187.7 (186.6, 188.7)	185.9 (184.8, 187.0)	185.8 (184.6, 187.1)	185.5 (184.7, 186.3)	186.1 (185.2, 186.9)	185.9 (185.4, 186.4)	186.5 (185.9, 187.1)	0.29


*Notes*: All values are in column percentages and 95% confidence interval unless specified; MET minutes – metabolic equivalents minutes; * Age and sex standardized to 2010 South Asia population. #Chi square test for categorical variables and One-way ANOVA for means of the standardized estimates.

The age and sex standardized mean number of ideal cardiac metrics were 3.7, 3.3, 3.6, and 4.1 in overall, metropolitan, smaller cities, and rural areas, respectively. Only 0.2% met all seven ideal cardiac health metrics. None of the ideal cardiac health metrics were present in 0.3%. (Table 4).

**Table 4:** Prevalence of ideal cardiac health metrics in three levels of residence in India.


NUMBER OF IDEAL CARDIAC HEALTH METRICS#	METROPOLITAN CITIES		SMALLER CITIES		RURAL		TOTAL	
			
	n = 7,106		n = 4,948		n = 10,090		n = 22,144	
			
	CRUDE % (95% CI)	STANDARDIZED* % (95% CI)	CRUDE % (95% CI)	STANDARDIZED* % (95% CI)	CRUDE % (95% CI)	STANDARDIZED* % (95% CI)	CRUDE % (95% CI)	STANDARDIZED* % (95% CI)

**0**	0.5 (0.3, 0.7)	0.5 (0.3, 0.7)	0.4 (0.3, 0.7)	0.5 (0.3, 0.7)	0.1 (0.1, 0.2)	0.2 (0.1, 0.3)	0.3 (0.2, 0.4)	0.3 (0.3, 0.4)

**Any 1**	5.5 (5.0, 6.1)	6.7 (5.9, 7.4)	4.5 (3.9, 5.1)	5.0 (4.3, 5.7)	1.6 (1.3, 1.8)	1.8 (1.5, 2.2)	3.5 (3.3, 3.7)	4.0 (3.7, 4.3)

**Any 2**	18.8 (17.9, 19.7)	21.4 (20.2, 22.6)	15.3 (14.3, 16.3)	16.8 (15.6, 18.0)	7.4 (6.9, 7.9)	8.1 (7.5, 8.7)	12.8 (12.4, 13.2)	14.0 (13.5, 14.6)

**Any 3**	27.6 (26.5, 28.6)	27.7 (26.5, 28.9)	24.8 (23.6, 26.0)	25.6 (24.3, 27.0)	19.4 (18.7, 20.2)	20.8 (19.9, 21.6)	23.2 (22.7, 23.8)	24.2 (23.6, 24.8)

**Any 4**	25.5 (24.5, 26.5)	23.9 (22.8, 25.0)	26.3 (25.0, 27.5)	26.2 (24.8, 27.5)	29.7 (28.8, 30.6)	30.2 (29.2, 31.2)	27.6 (27.0, 28.2)	27.5 (26.9, 28.2)

**Any 5**	15.8 (15.0,16.7)	14.2 (13.3, 15.1)	19.3 (18.3, 20.5)	17.6 (16.5, 18.7)	27.8 (27.0, 28.7)	26.9 (26.0, 27.8)	22.1 (21.5, 22.6)	20.8 (20.2, 21.4)

**Any 6**	6.4 (5.8, 7.0)	5.6 (5.1, 6.2)	8.9 (8.1, 9.7)	7.9 (7.2, 8.6)	13.8 (13.1, 14.5)	11.8 (11.2, 12.4)	10.3 (9.9, 10.7)	8.9 (8.6, 9.3)

**Any 7**	0.0 (0.0, 0.1)	0.0 (0.0, 0.0)	0.5 (0.4, 0.8)	0.4 (0.2, 0.6)	0.2 (0.1, 0.3)	0.2 (0.1, 0.3)	0.2 (0.2, 0.3)	0.2 (0.1, 0.3)

**Mean (SD)**	3.5 (3.4, 3.5)	3.3 (3.3, 3.3)	3.7 (3.6, 3.7)	3.6 (3.6, 3.6)	4.2 (4.1, 4.2)	4.1 (4.1, 4.1)	3.8 (3.8, 3.8)	3.7 (3.7, 3.8)


*Notes*:
The ideal CVH metrics included are not smoking, diet, physical activity, body mass index, blood pressure, fasting plasma glucose and total cholesterol. All values are in column percentage (95% confidence interval) except when specified. # mutually exclusive categories; * Age and sex standardized to 2010 South Asia population. The differences number of ideal CVH across the three types of settings and mean metrics were significant at p-value <0.001 for both crude and standardized estimates using the chi-square test and one way ANOVA, respectively.

The overall prevalence of good, moderate, and poor CVH were 9.9%, 48.9%, and 41.2% respectively. The distribution of good, moderate, and poor CVH stratified by residence were 3.9%, 41.0%, and 55.1% for metropolitan cities; 7.8%, 49.2% (95%CI: 47.8%, 50.7%), and 43.0% for smaller cities; and 10.4%, 60.9%, and 28.7% in rural villages, respectively, after adjusting for age, sex, education, and asset tertiles. (E-table 1)

**Figure 2:**
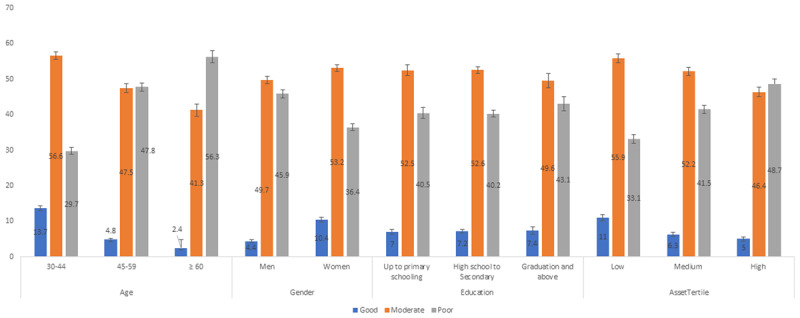
Adjusted prevalence of good, moderate and poor cardiovascular health by socio-demographic factors (N = 22,144). *Notes*: The bars show precentages and line represents 95% confidence intervals. * Adjusted for age, sex, education, asset index and place of residence.

**Figure 3:**
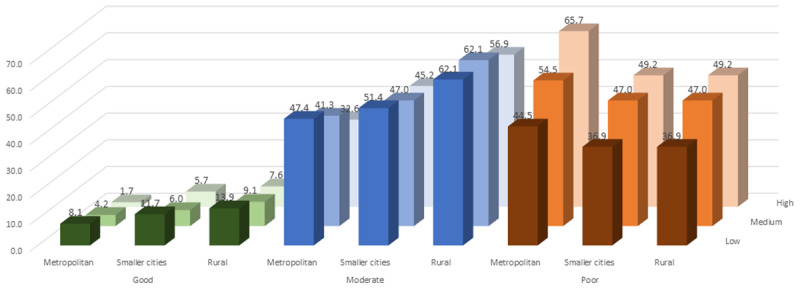
Adjusted prevalence of good, moderate and poor cardiovascular health by asset index in Metropolitan cities, smaller cities, and rural areas. Note: The bars show percentages. *Adjusted for age, sex, and education.

The adjusted prevalence (predicted marginals) of good cardiac health was higher in younger age groups: 30–44 years (13.7%), 45–59 years (4.8%), and ≥60 years (2.4%), respectively. The adjusted prevalence of good CVH was lower in men (4.4%) compared to women (10.4%), and good CVH did not vary by education but was lowest in the high asset tertile (11.0%) compared to middle (6.3%) and low tertiles (5.0%) (Figure 2). The adjusted prevalence of good, moderate, and poor CVH by asset tertiles in metropolitan, smaller cities, and rural areas are shown in Figure 3. There was a gradient of CVH across the asset tertiles seen in these three types of settings.

Sensitivity analyses comparing findings from analytical dataset (N = 22,144) and complete dataset including those with missing outcome data (N = 27,051) showed little differences in the distribution of age, sex, education, and wealth index (E-table 2) and estimated ideal CVH metrics (estimated from IPW in completed dataset) (E-table 3). When participants who were on medication for diabetes, hypertension, and dyslipidemia were reclassified as having poor CVH, we found little differences in our estimates (E-table 4)

## Discussion

Benchmarking and reporting on ideal CVH metrics is a practical strategy in communities and identifies areas that require further attention and improvement. In this pooled analysis of individual-level data on ideal CVH metrics in metropolitan, smaller cities, and rural populations of India, we noted poor achievement of CVH overall and inverse relationships between urbanization and CVH health, and between socioeconomic status and CVH health. On average, less than four of the seven goals were met with slight variation between cities and rural areas. Nearly no one in the study population met all seven ideal CVH goals. Good CVH (≥ 6 ideal metrics) was present in a small proportion of the population (ranging from 4% in metropolitan to 10% in rural areas). While younger adults fared better than older adults, only one in five young adults in rural areas to just one in twelve young adults in metropolitan cities achieved this level of CVH.

In an earlier study of 11 medium-sized cities in India, similarly low levels of good CVH were reported. The authors noted 3.5% and 3.6% of adult men and women, respectively, achieved good CVH [9]. In our study, good CVH, even in rural areas, was far lower than global estimates of 19% noted in a sub-analysis of 55 studies in a systematic review across the globe [8]. The distribution of CVH metrics in our study across sex and age groups were similar to previous findings [8].

Aligned with other studies [8,9], not smoking was the most common ideal CVH metric achieved in our study, and diet, the least. City dwellers performed better in non-smoking compared with rural areas. These differences would be even more striking if smokeless tobacco use was included as its use is higher in rural areas [18]. The ideal diet metric in our study, although simple and measurable, provides a very narrow perspective on healthy diet. To date, there is no single measure that uniformly and comprehensively conveys the amount and quality of total calories consumed [4]. As such, this dietary indicator may not fully capture the breadth and nutrient content of foods consumed in India.

We found varied relationships between education and asset tertiles with ideal CVH metrics across settings. There was little variation of good CVH metrics across education groups in all three settings. While education represents socioeconomic position in terms of earning potential, it also provides a window into literacy, knowledge, and cognitive functioning [19]. It stands, therefore, that education tends to correlate with better health-related behavior [19,20]. This has been documented in past reports from CARRS [13] and SSS [12], that lower education was associated with higher prevalence of smoking and low fruits and vegetable intake. In contrast, higher-educated individuals tended to have abnormal metabolic factors (obesity, high glucose, and high cholesterol) [12,13]. When behavioral and metabolic factors were combined together as in the ideal CVH metrics, we found no variation across education categories. On the contrary, we found a significant gradient in CVH metrics across tertiles of asset index in all three settings with the poorest demonstrating better CVH in all settings; this finding has been noted previously [9]. Still, the gradients across metropolitan, smaller city, and rural areas were more prominent than the gradient across asset tertiles. Participants from lowest asset tertiles of metropolitan cities had a higher prevalence (44.7%) of poor CVH (≤3 factors) when compared to the highest tertile of rural areas. This underscores the stronger association between urban residence and poor CVH.

India is one of the fastest-growing economies in the world. A large proportion of people are moving to cities for jobs and better prospects. Peri-urban parts of the cities are increasingly urbanized. Urban living is associated with ease of access to energy-dense, unhealthy foods, higher use of motorized transports, mechanized work, mental stress, low access to leisure-time physical activity, and environmental stressors such as air pollution. Therefore, urban dwellers, specifically the urban poor, are more susceptible to CVD, and should be a focus going forward. Further, migrants from rural to urban areas acquire urban level risk factors [21]. Ongoing efforts by the Government of India such as Smart Cities Mission [22] and the National Multisectoral Action Plan for prevention and control of common non-communicable diseases may need to target the highest risk groups and design implementation strategies that optimize the motivations, capabilities, and priorities of the urban poor [23].

Our study had a few limitations which need to be considered while interpreting the findings. The surveys used different physical activity questionnaires: CARRS and SSS used IPAQ and the UDAY study used GPAQ. The IPAQ measures the physical activity of the previous week while GPAQ measures the activity in a typical week. In a validation study of GPAQ and IPAQ against a gold standard (accelerometry), both questionnaires were found to be comparable [15,24] and both overestimate physical activity [24]. The actual physical activity level in the community was likely to be much lower than the estimated levels, and therefore, the overall ideal CVH levels may be lower in reality than the estimates reported in this study. Additionally, we have stratified participants into metropolitan, smaller city, and rural areas based on their residence during the survey, but the duration of their stay in these cities and villages was not measured and accounted for in the analysis.

The study did have several strengths. We used individual-level data from three large, representative household samples from diverse settings in India. Although we had excluded 4,907 data points due to missing variables, the sensitivity analysis showed no difference between the complete and included datasets. We used standardized questionnaires, physical measurement, and laboratory methods across settings.

## Conclusion

Ideal CVH metrics are dismally low in both large and smaller cities and rural villages in India, with less than 1 in 10 people achieving ≥6 or more markers. Women, younger adults, the least wealthy, and rural residents had significantly higher achievement of good CVH compared to their counterparts. This has major implications for local and national leaders in how to improve CVH in India. Multisectoral actions are imperative to improve cardiac health, and specifically in cities.

## Additional Files

The additional files for this article can be found as follows:

10.5334/gh.1137.s1 E-table 1. Unadjusted and adjusted prevalence of poor, moderate and good cardiac health by sociodemographic characteristics in India.

10.5334/gh.1137.s2 E-table 2. Sensitivity analysis- comparison of sociodemographic variables of the included and complete dataset.

10.5334/gh.1137.s3 E-table 3. Sensitivity analysis- estimated individual ideal cardiac health metrics in the complete dataset using inverse probability weighting.

10.5334/gh.1137.s4 E-table 4. Comparison of Ideal CVH definition disregard to treatment status and participants on medication stratified as ‘poor’.
