# Correction: Cardiovascular Health in India – a Report Card from Three Urban and Rural Surveys of 22,144 Adults

**DOI:** 10.5334/gh.1158

**Published:** 2022-09-22

**Authors:** Roopa Shivashankar, Kalpana Singh, Dimple Kondal, Ruby Gupta, Pablo Perel, Deksha Kapoor, Devraj Jindal, Sailesh Mohan, Rajendra Pradeepa, Prashant Jarhyan, Nikhil Srinivasapura Venkateshmurthy, Nikhil Tandon, Viswanathan Mohan, K. M. Venkat Narayan, Dorairaj Prabhakaran, Mohammed K. Ali

**Affiliations:** 1 Indian Council of Medical Research (ICMR), New Delhi, IN; 2 Centre for Chronic Disease Control (CCDC), New Delhi, IN; 3 Hamad Medical Corporation, Doha, QA; 4 Public Health Foundation of India (PHFI), New Delhi, IN; 5 London School of Hygiene and Tropical Medicine (LSHTM), London, GB; 6 All India Institute of Medical Sciences (AIIMS), New Delhi, IN; 7 Global Academy of Agriculture and Food Systems, University of Edinburgh, Edinburgh, GB; 8 Deakin University, Melbourne, AU; 9 Madras Diabetes Research Foundation (MDRF), Chennai, IN; 10 Rollins School of Public Health & Emory Global Diabetes Research Center, Emory University, Atlanta, US; 11 Rollins School of Public Health, Emory University, Atlanta, US; 12 Rollins School of Public Health & Department of Family and Preventive Medicine, School of Medicine, Emory University, Atlanta, US

**Keywords:** Cardiology, Cardiovascular Health, Global Health, India

## Abstract

This article details a correction to: Shivashankar R, Singh K, Kondal D, Gupta R, Perel P, Kapoor D, et al.. Cardiovascular Health in India – a Report Card from Three Urban and Rural Surveys of 22,144 Adults. Global Heart. 2022; 17(1): 52. DOI: http://doi.org/10.5334/gh.1137

## Correction

After the publication of ‘Cardiovascular Health in India – a Report Card from Three Urban and Rural Surveys of 22,144 Adults’ [1], the authors noted an error in Figure 3 of the paper.

They had inadvertently repeated the percentage of poor cardiac health in low, middle and high wealth index groups of smaller cities in the rural area. The correct percentage of poor cardiac health in low, middle, and high wealth index groups of rural areas are 24.0%, 28.8%, and 35.5% respectively (Ref: Figure 3).

**Figure 3:**
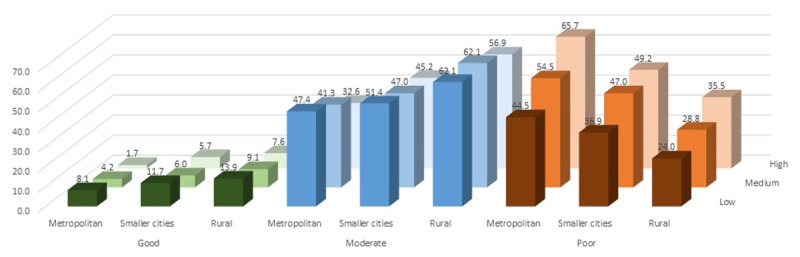
Adjusted prevalence of good, moderate and poor cardiovascular health by asset index in Metropolitan cities, smaller cities, and rural areas. Note: The bars show percentages. * Adjusted for age, sex, and education.

In the original, incorrect figure, these percentages were listed as 36.9%, 47.0%, and 49.2%.
