# Supplementary material for: Cardiovascular Health in India – a Report Card from Three Urban and Rural Surveys of 22,144 Adults
Source: Glob Heart. 2022 Aug 2;17(1):52. doi: 10.5334/gh.1137 (PMC9354560; doi:10.5334/gh.1137)
Supplement: E-table 1. — Unadjusted and adjusted prevalence of poor, moderate and good cardiac health by sociodemographic characteristics in India. [file gh-17-1-1137-s1.pdf]

etable 1: Unadjusted and adjusted prevalence of poor, moderate and good cardiac health by sociodemographic characteristics in India

		Metropolitan Cities % (95% CI)			Smaller cities % (95% CI)			Rural % (95% CI)			Total % (95% CI)		
		**Good**	**Moderate**	**Poor**	**Good**	**Moderate**	**Poor**	**Good**	**Moderate**	**Poor**	**Good**	**Moderate**	**Poor**
All	uadjusted	6.2 (5.7,6.8)	40.9 (39.7,42.0)	52.9 (51.8,54.1)	9.3 (8.5,10.1)	45.3 (43.9,46.6)	45.4 (44.1,46.8)	13.8 (13.2,14.5)	57.3 (56.4,58.3)	28.8 (28.0,29.7)	10.4 (9.7, 11.2)	49.4 (46.8 , 51.9)	40.2 (37.2, 43.2)
	adjusted	**3.9 (3.5,4.3)**	**41.0 (39.8,42.1)**	**55.1 (53.9,56.3)**	**7.8 (7.0,8.6)**	**49.2 (47.8,50.7)**	**43.0 (41.5,44.4)**	**10.4 (9.7,11.1)**	**60.9 (59.9,62.0)**	**28.7 (27.7,29.7)**	**7.2 (6.7,7.6)**	**52.1 (51.4,52.8)**	**40.7 (40.0,41.5)**
30-44	uadjusted	9.9 (9.0,10.9)	48.7 (47.1,50.4)	41.4 (39.8,43.0)	15.2 (13.8,16.7)	52.0 (50.0,54.0)	32.8 (31.0,34.8)	22.7 (21.5,23.9)	58.7 (57.3,60.1)	18.6 (17.5,19.8)	15.8 (15.2,16.5)	53.8 (52.9,54.7)	30.4 (29.6,31.2)
	adjusted	7.8 (6.8,8.7)	48.9 (47.2,50.6)	43.3 (41.6,45.0)	13.4 (12.0,14.9)	53.0 (50.9,55.1)	33.6 (31.6,35.6)	19.5 (18.2,20.9)	61.2 (59.6,62.8)	19.3 (18.0,20.6)	13.7 (13.0,14.4)	56.6 (55.5,57.6)	29.7 (28.8,30.7)
45-59	uadjusted	2.8 (2.2,3.5)	35.3 (33.5,37.2)	61.9 (60.0,63.8)	5.5 (4.5,6.6)	40.9 (38.6,43.2)	53.7 (51.3,56.0)	8.2 (7.3,9.2)	58.5 (56.8,60.1)	33.3 (31.8,34.9)	5.5 (5.0,6.0)	46.3 (45.2,47.3)	48.3 (47.2,49.3)
	adjusted	2.5 (1.9,3.1)	36.3 (34.4,38.2)	61.2 (59.3,63.1)	4.8 (3.8,5.8)	41.3 (39.0,43.7)	53.9 (51.5,56.3)	7.2 (6.3,8.2)	59.0 (57.2,60.8)	33.8 (32.1,35.5)	4.8 (4.3,5.2)	47.5 (46.3,48.7)	47.8 (46.6,49.0)
≥ 60	uadjusted	1.9 (1.3,2.9)	27.4 (24.9,30.1)	70.7 (67.9,73.3)	1.9 (1.2,3.0)	36.8 (33.8,39.9)	61.3 (58.2,64.4)	3.9 (3.1,4.8)	52.5 (50.4,54.6)	43.6 (41.5,45.7)	2.7 (2.3,3.2)	41.4 (40.0,42.9)	55.9 (54.4,57.3)
	adjusted	1.9 (1.1,2.7)	28.6 (25.8,31.4)	69.5 (66.7,72.4)	1.8 (0.99,2.7)	37.7 (34.5,40.9)	60.5 (57.2,63.7)	3.6 (2.8,4.5)	53.6 (51.2,56.1)	42.7 (40.3,45.1)	2.4 (1.9,2.8)	41.3 (39.6,42.9)	56.3 (54.7,58.02)
Men	uadjusted	4.1 (3.5,4.8)	37.6 (36.0,39.3)	58.3 (56.6,59.9)	5.2 (4.3,6.1)	40.4 (38.4,42.4)	54.4 (52.4,56.4)	7.1 (6.4,7.9)	59.6 (58.2,61.1)	33.2 (31.9,34.7)	5.4 (5.0,5.8)	47.3 (46.4,48.3)	47.3 (46.4,48.2)
	adjusted	3.0 (2.5,3.6)	38.8 (37.1,40.5)	58.2 (56.4,60.0)	4.1 (3.3,4.9)	42.0 (39.9,44.1)	53.9 (51.8,56.0)	6.0 (5.2,6.8)	62.0 (60.3,63.6)	32.0 (30.4,33.6)	4.4 (4.0,4.8)	49.7 (48.7,50.8)	45.9 (44.8,47.0)
Women	uadjusted	8.0 (7.2,8.9)	43.6 (42.1,45.1)	48.4 (46.9,50.0)	12.9 (11.7,14.2)	49.6 (47.7,51.4)	37.5 (35.7,39.3)	18.8 (17.8,19.8)	55.6 (54.3,56.9)	25.6 (24.5,26.7)	13.5 (13.0,14.1)	50.2 (49.4,51.1)	36.2 (35.4,37.1)
	adjusted	5.9 (5.1,6.7)	44.1 (42.5,45.7)	50.0 (48.4,51.7)	10.0 (8.7,11.3)	51.2 (49.1,53.2)	38.9 (36.9,40.9)	15.0 (13.9,16.0)	59.4 (57.9,60.8)	25.7 (24.4,27.0)	10.4 (9.8,11.1)	53.2 (52.2,54.1)	36.4 (35.5,37.4)
Up to primary schooling	uadjusted	6.1 (5.0,7.4)	43.5 (41.0,46.0)	50.4 (47.9,52.9)	11.2 (9.6,13.1)	49.8 (47.0,52.6)	39.0 (36.3,41.7)	13.9 (12.8,15.0)	56.7 (55.2,58.2)	29.4 (28.0,30.9)	11.1 (10.4,11.9)	51.9 (50.8,53.0)	37.0 (35.9,38.1)
	adjusted	3.5 (2.7,4.4)	42.5 (39.8,45.3)	54.0 (51.2,56.7)	6.3 (4.9,7.8)	49.7 (46.5,52.9)	44.0 (40.7,47.3)	10.8 (9.5,12.0)	60.8 (58.9,62.8)	28.4 (26.5,30.3)	7.0 (6.3,7.7)	52.5 (51.0,54.0)	40.5 (39.0,42.0)
High school to Secondary	uadjusted	6.9 (6.2,7.6)	42.4 (41.0,43.8)	50.8 (49.3,52.2)	9.4 (8.3,10.7)	45.3 (43.3,47.3)	45.3 (43.3,47.3)	14 (13.1,14.9)	57.6 (56.3,58.9)	28.4 (27.2,29.6)	10 (9.5,10.5)	49.2 (48.4,50.1)	40.8 (40.0,41.6)
	adjusted	4.5 (3.8,5.2)	42.6 (41.1,44.1)	52.9 (51.4,54.5)	7.0 (5.9,8.0)	47.5 (45.4,49.6)	45.6 (43.5,47.7)	10.0 (9.1,10.9)	61.3 (59.9,62.7)	28.8 (27.5,30.1)	7.2 (6.7,7.7)	52.6 (51.6,53.5)	40.2 (39.3,41.2)
Graduation and above	uadjusted	3.8 (2.9,5.1)	31.7 (29.1,34.4)	64.5 (61.8,67.2)	7.4 (6.1,8.9)	41.2 (38.6,43.8)	51.4 (48.8,54.0)	12.5 (10.2,15.3)	58.4 (54.6,62.1)	29.1 (25.8,32.7)	6.9 (6.1,7.7)	41.5 (39.9,43.1)	51.6 (50.0,53.3)
	adjusted	4.9 (3.4,6.4)	37.8 (34.6,41.0)	57.3 (54.0,60.6)	6.6 (5.1,8.0)	45.1 (42.2,48.1)	48.3 (45.3,51.3)	11.2 (8.7,13.8)	61.8 (57.9,65.7)	27.0 (23.4,30.6)	7.4 (6.3,8.4)	49.6 (47.6,51.5)	43.1 (41.1,45.1)
Low	uadjusted	10.0 (8.9,11.2)	47.9 (46.0,49.8)	42.1 (40.3,44.0)	16.2 (14.0,18.6)	50.5 (47.4,53.6)	33.3 (30.4,36.3)	17.9 (16.7,19.2)	58.3 (56.7,60.0)	23.8 (22.4,25.2)	14.4 (13.6,15.2)	53.2 (52.1,54.3)	32.4 (31.3,33.5)
	adjusted	8.1 (6.9,9.2)	47.4 (45.4,49.4)	44.5 (42.5,46.5)	11.7 (9.4,14.0)	51.4 (47.8,54.9)	36.9 (33.4,40.4)	13.9 (12.6,15.2)	62.1 (60.4,63.9)	24.0 (22.4,25.5)	11.0 (10.2,11.8)	55.9 (54.6,57.2)	33.1 (31.9,34.4)

Medium	uadjusted	5.3 (4.5,6.2)	41.3 (39.5,43.2)	53.4 (51.5,55.2)	8.1 (6.9,9.4)	45.5 (43.3,47.8)	46.4 (44.1,48.7)	11.4 (10.4,12.6)	59.0 (57.3,60.7)	29.6 (28.0,31.2)	8.1 (7.6,8.7)	49.2 (48.1,50.2)	42.7 (41.7,43.7)
	adjusted	4.2 (3.5,5.0)	41.3 (39.39,43.2)	54.5 (52.6,56.4)	6.0 (4.9,7.1)	47.0 (44.7,49.4)	47.0 (44.6,49.4)	9.1 (8.0,10.1)	62.1 (60.4,63.8)	28.8 (27.2,30.5)	6.3 (5.8,6.9)	52.2 (51.0,53.3)	41.5 (40.4,42.7)
High	uadjusted	1.9 (1.4,2.6)	29.5 (27.5,31.7)	68.6 (66.4,70.6)	7.2 (6.1,8.3)	42.1 (40.0,44.2)	50.7 (48.6,52.9)	9.7 (8.5,11.0)	54.3 (52.2,56.3)	36.1 (34.1,38.1)	6.1 (5.6,6.7)	42.6 (41.5,43.8)	51.3 (50.1,52.4)
	adjusted	1.7 (1.1,2.3)	32.6 (30.2,35.1)	65.7 (63.2,68.2)	5.7 (4.6,6.7)	45.2 (42.9,47.5)	49.2 (46.8,51.5)	7.6 (6.5,8.7)	56.9 (54.7,59.0)	35.5 (33.4,37.6)	5.0 (4.4,5.5)	46.4 (45.0,47.7)	48.7 (47.3,50.1)

Notes: All adjusted figures are average predicted marginals after adjusting for other demographic variables - age, gender, education, asset tertiles and sites
