# Supplementary material for: Cardiovascular Health in India – a Report Card from Three Urban and Rural Surveys of 22,144 Adults
Source: Glob Heart. 2022 Aug 2;17(1):52. doi: 10.5334/gh.1137 (PMC9354560; doi:10.5334/gh.1137)
Supplement: E-table 2. — Sensitivity analysis- comparison of sociodemographic variables of the included and complete dataset. [file gh-17-1-1137-s2.pdf]

etable 2: Sensitivity analysis- comparison of sociodemographic variables of the included and complete dataset

	CARRS					Uday					SSS					Total				
	Included dataset		Complete dataset*		p value#	Included dataset		Complete dataset*		p value#	Included dataset		Complete dataset*		p value#	Included dataset		Complete dataset*		p value#
	n= 7106		n=9854			n= 4948		n=5851			n=10,090		n=11346			n=22,144		n=27,051		
	%	95% CI	%	95% CI		%	95% CI	%	95% CI		%	95% CI	%	95% CI		%	95% CI	%	95% CI	
**Age in years (Mean)**	45.8	(45.6, 46.0)	45.8	(45.6, 46.0)	0.990	47	(46.7, 47.3)	47	(46.7, 47.3)	0.990	47.6	(47.4, 47.9)	47.6	(47.4, 47.9)	0.990	46.8	(46.7, 47.0)	46.8	(46.7, 47.0)	0.990
**Sex (%)**																				
Male	45.2	(44.1, 46.4)	48.1	(47.1, 49.1)	0.002	46.3	(44.9, 47.7)	46.2	(44.9, 47.5)	0.920	42.1	(41.2, 43.1)	42.8	(41.9, 43.7)	0.299	44.0	(43.4, 44.7)	45.5	(44.9, 46.1)	0.009
Female	54.8	(53.6, 55.9)	51.9	(50.9, 52.9)	0.002	53.7	(52.3, 55.1)	53.8	(52.5, 55.1)	0.970	57.9	(56.9, 58.8)	57.2	(56.3, 58.1)	0.299	56.0	(55.3, 56.6)	54.5	(53.9, 55.1)	0.009
**Education (%)**																				
Primary	20.3	(19.4, 21.3)	20.8	(20.0, 21.6)	0.426	24.9	(23.7, 26.1)	25.3	(24.2, 26.5)	0.630	38.9	(38.0, 39.9)	40	(39.1, 40.9)	0.764	29.8	(29.2, 30.4)	29.8	(29.3, 30.4)	0.990
High school	63.2	(62.1, 64.4)	62.6	(61.6, 63.5)	0.424	47.6	(46.2, 49.0)	47.1	(45.8, 48.4)	0.604	54.6	(53.6, 55.5)	53.8	(52.9, 54.7)	0.239	55.8	(55.1, 56.5)	55.5	(54.9, 56.1)	0.510
Graduation	16.4	(15.6, 17.3)	16.7	(16.0, 17.4)	0.426	27.5	(26.3, 28.8)	27.6	(26.4, 28.7)	0.421	6.5	(6.1, 7.0)	6.2	(5.8, 6.7)	0.367	14.4	(13.9, 14.9)	14.7	(14.2, 15.1)	0.350
**Asset Tertiles (%)**																				
Low	37.9	(36.7, 39.0)	39.1	(38.1, 40.0)	0.113	21.5	(20.4, 22.7)	22.1	(21.1, 23.2)	0.350	46.1	(45.1, 47.1)	46.9	(46.0, 47.8)	0.240	38.0	(37.3, 38.6)	38.7	(38.1, 39.3)	0.112
Middle	20.5	(19.6, 21.5)	20.2	(19.4, 21.0)	0.631	43.1	(41.7, 44.5)	42.6	(41.3, 43.8)	0.600	35.3	(34.4, 36.2)	35.3	(34.4, 36.2)	0.990	32.3	(31.7, 32.9)	31.4	(30.8, 31.9)	0.030
High	41.6	(40.5, 42.8)	40.7	(39.8, 41.7)	0.239	35.4	(34.1, 36.7)	35.3	(34.1, 36.5)	0.890	18.6	(17.9, 19.4)	17.8	(17.1, 18.5)	0.180	29.7	(29.1, 30.3)	29.9	(29.4, 30.5)	0.450

Notes:
*Included 4907 participants who were excluded due to missing values in one or more ideal CVH metrics;
using chi-square test for comparison of categories and t-test for comparison of means
