# Supplementary material for: Cardiovascular Health in India – a Report Card from Three Urban and Rural Surveys of 22,144 Adults
Source: Glob Heart. 2022 Aug 2;17(1):52. doi: 10.5334/gh.1137 (PMC9354560; doi:10.5334/gh.1137)
Supplement: E-table 3. — Sensitivity analysis- estimated individual ideal cardiac health metrics in the complete dataset using inverse probability weighting. [file gh-17-1-1137-s3.pdf]

etable 3: Sensitivity analysis- estimated individual ideal cardiac health metrics in the complete dataset using inverse probability weighting*

Variables	Definition	Metropolitan cities n=9,854	Smaller cities n=5,851	Rural n=11,346	Total n=27,051
**Smoking**					
Ideal	Non-smoker	83.4 (82.7, 84.2)	81.6 (80.5, 82.5)	71.6 (70.7, 72.4)	78.1 (77.5,78.5)
Intermediate	Past smoker	0.0 (0.0, 0.1)	0.0 (0.0, 0.0)	0.0 (0.0,0.0)	0.0 (0.0, 0.0)
Poor	Current smoker	16.5(15.8, 17.3)	18.4 (17.5, 19.5)	28.4 (27.6, 29.3)	21.9 (21.5, 22.4)
**Diet (fruits and vegetable consumption)**					
Ideal	≥ 5 servings	0.6 (0.4, 0.7)	11.9 (11.1, 12.8)	3.2 (2.9,3.6)	4.1 (3.9, 4.4)
Intermediate	2-4 servings	45.4 (44.4, 46.4)	51.5 (50.2, 52.8)	41.3 (40.4, 42.2)	45.0 (44.4, 45.6)
Poor	<2 servings	54.0 (53.0, 55.0)	36.6 (35.3, 37.8)	55.5 (54.6, 56.4)	50.9 (50.3, 51.5)
Mean servings		1.5 (1.5, 1.5)	2.9 (2.8, 2.9)	2.1 (2.1, 2.1)	2.0 (2.0, 2.1)
**Physical activity**					
Ideal	High activity	65.8 (64.9, 66.8)	57.4 (56.2, 58.7)	79.8 (79.1, 80.5)	69.9 (69.3, 70.4)
Intermediate	Moderate activity	26.2 (25.3, 27.1)	26.2 (25.1, 27.3)	11.5 (10.9, 12.1)	20.0 (19.5, 20.5)
Poor	Low activity	8.0 (7.5, 8.5)	16.4 (15.5, 17.4)	8.7 (8.2, 9.2)	10.1 (9.7, 10.5)
Mean MET minutes		4533.7 (4473.4, 4594.0)	5651.3 (5490.1, 5812.5)	9518.7 (9390.7, 9646.7)	6869.2 (6796.1, 6942.2)
**Blood Pressure (BP in mm/Hg)**					
Ideal	<120/80	32.2 (31.3, 33.2)	32.2 (31.3, 33.2)	32.2 (31.3, 33.2)	37.5 (36.9, 38.1)
Intermediate	120-139/ 80-89	37.9 (36.9, 38.9)	37.9 (36.9, 38.9)	37.9 (36.9, 38.9)	37.9 (37.3, 38.5)
Poor	≥140/90	29.9 (29.0, 30.9)	29.9 (29.0, 30.9)	29.9 (29.0, 30.9)	24.6 (24.1, 25.1)
Mean Systolic BP		125.7 (125.3, 126.1)	126.7 (126.2, 127.2)	123.8 (123.5, 124.2)	125.1 (124.9, 125.4)
Mean Diastolic BP		83.2 (83.0, 83.4)	77.4 (77.1, 77.7)	76.7 (76.5, 77.0)	79.2 (79.0, 79.3)
**Body mass index (BMI in kg/m2)**					
Ideal	<25.0	45.5 (44.4, 46.6)	47.3 (46.0, 48.6)	72.6 (71.8, 73.5)	58.3 (57.6, 58.9)
Intermediate	25.0-29.9	35.6 (34.5, 36.7)	35.9 (34.6, 37.1)	21.1 (20.4, 21.9)	29.1 (28.5, 29.7)

Poor	≥ 30	18.9 (18.1, 19.8)	16.8 (15.9, 17.8)	6.2 (5.8, 6.7)	12.6 (12.2, 13.1)
Mean BMI		25.8 (25.7, 25.9)	25.6 (25.5, 25.8)	22.8 (22.7, 22.9)	24.4 (24.4, 24.5)
**Fasting plasma glucose (FPG in mg/dl)**					
Ideal	<100	51.1 (50.1, 52.2)	65.6 (64.2, 66.9)	78.4 (77.6, 79.2)	66.1 (65.5, 66.7)
Intermediate	100-125	32.0 (31.0, 33.0)	20.8 (19.7, 21.9)	16.3 (15.6, 17.0)	22.8 (22.2, 23.3)
Poor	≥126	16.9 (16.1, 17.7)	13.7 (12.8, 14.7)	5.3 (4.9, 5.7)	11.2 (10.8, 11.6)
Mean FPG		113.6 (112.6, 114.6)	104.0 (102.7, 105.2)	94.5 (93.9, 95.1)	103.2 (102.7, 103.7)
**Total Cholesterol (TC in mg/dl)**					
Ideal	<200	66.2 (65.2, 67.3)	66.1 (64.8, 67.4)	66.5 (65.6, 67.4)	66.4 (65.7, 67.0)
Intermediate	200-239	24.9 (24.0, 25.9)	24.5 (23.3, 25.7)	23.6 (22.7, 24.4)	24.2 (23.7, 24.8)
Poor	≥240	8.8 (8.2, 9.5)	9.4 (8.6, 10.2)	9.9 (9.3, 10.5)	9.4 (9.0, 9.8)
Mean TC		186.4 (185.6, 187.2)	185.9 (184.8, 187.0)	185.4(184.6, 186.2)	185.9 (185.4, 186.4)

*The complete dataset includes the 4907 participants who were excluded due to missing values in one or more ideal CVH metrics; estimates from inverse probability weighting using age, sex, education and wealth index variables**.**
