# Supplementary material for: Cardiovascular Health in India – a Report Card from Three Urban and Rural Surveys of 22,144 Adults
Source: Glob Heart. 2022 Aug 2;17(1):52. doi: 10.5334/gh.1137 (PMC9354560; doi:10.5334/gh.1137)
Supplement: E-table 4. — Comparison of Ideal CVH definition disregard to treatment status and participants on medication stratified as ‘poor’. [file gh-17-1-1137-s4.pdf]

etable 4: Comparison of Ideal CVH definition disregard to treatment status and participants on medication stratified as ‘poor’ (N= 22144)

Ideal factors	CVH definition disregard to treatment status	Participants on medication* stratified as ‘poor’ CVH
Ideal Blood Pressure	34.5 (33.9, 35.2)	32.9 (32.3, 33.5)
Ideal FPG	65.8 (65.1, 66.5)	64.5(63.8, 65.1)
Ideal Total Cholesterol	65.4 (64.7, 66.1)	65.2(64.5, 65.9)
Ideal CVH scores		
0	0.3 (0.3, 0.4)	0.4 (0.3, 0.5)
1	4.0 (3.7, 4.3)	4.4 (4.1, 4.7)
2	14.0 (13.5,14.6)	15.2 (14.7, 15.7)
3	24.2 (23.6, 24.8)	24.6 (24.0, 25.2)
4	27.5 (26.9, 28.2)	26.9 (26.3, 27.5)
5	20.8 (20.2, 21.4)	20.0 (19.5, 20.6)
6	8.9 (8.6, 9.3)	8.3 (8.0, 8.7)
7	0.2 (0.1, 0.3)	0.2 (0.1, 0.2)
Mean	3.7 (3.7, 3.8)	3.7 (3.7, 3.7)
Good CVH	8.7 (8.4, 9.1)	8.5 (8.2, 8.9)
Moderate CVH	47.9(47.2, 48.6)	46.9 (46.2, 47.6)
Poor CVH	43.4 (42.7, 44.0)	44.6 (43.9, 45.3)

Notes: All values in Percentage (95% confidence interval) age sex standardized to 2010 South Asia Population; FPG= Fasting Plasma Glucose; CVH= Cardiovascular Health

* Participants self-reported taking allopathic medication for diabetes, hypertension and high cholesterol
